# IRS2/FOXO1 mitigates osteoarthritis by regulating chondrocyte autophagy and mitochondrial function

**DOI:** 10.1186/s10020-025-01346-8

**Published:** 2025-09-26

**Authors:** Chaoren Qin, Kai Chen, Yingchun Sun, Changjiang Wang, Yaohui Yu, Hao Zhu, Guoyou Zou

**Affiliations:** 1 The Yancheng Clinical College of Xuzhou Medical University, Yancheng City, Jiangsu Province China; 2 The First People’s Hospital of Yancheng, Yancheng City, Jiangsu Province China; 3 Nanjing First Hospital, Nanjing Medical University, Nanjing, Jiangsu China; 4 Liutao Central Health Clinic, Xiangshui County, Yancheng City, Jiangsu Province China

**Keywords:** Osteoarthritis, Mitophagy, IRS2, FOXO1

## Abstract

**Purpose:**

Osteoarthritis (OA) is a debilitating joint disease with no effective cure. This study investigates the role of Insulin Receptor Substrate 2 (IRS2) in OA and its potential as a therapeutic target.

**Methods:**

Transcriptomic analysis of OA-related datasets (GSE178557, GSE169077, GSE64394, GSE57218) was conducted to identify differentially expressed genes (DEGs), with KEGG pathway analysis highlighting the PI3K/AKT pathway. In vivo, the destabilization of the medial meniscus (DMM) OA mouse model was used to assess IRS2 expression through histology, qPCR, and Western blot. IRS2 was overexpressed in primary mouse chondrocytes via adenoviral transfection, with proliferation, apoptosis, and autophagy assessed by EdU, Annexin V/PI staining, and autophagy-related protein analysis. Adenovirus expressing Irs2 was injected intra-articularly into DMM mice, and cartilage integrity was assessed using histology and micro-CT.

**Results:**

IRS2 expression was significantly reduced in OA cartilage, correlating with PI3K/AKT pathway inhibition. IRS2 overexpression restored AKT activation, FOXO1 phosphorylation, and mitochondrial autophagy. Intra-articular IRS2 injection improved cartilage matrix integrity, reduced MMP13, and alleviated subchondral bone changes in DMM mice.

**Conclusion:**

IRS2 plays a key role in OA pathogenesis and targeting it may provide a promising therapeutic approach for OA.

**Supplementary Information:**

The online version contains supplementary material available at 10.1186/s10020-025-01346-8.

## Introduction

Osteoarthritis (OA) is a chronic joint disease, affecting over 240 million people worldwide, leading to prolonged pain and functional impairments [1, 2]. This condition profoundly impacts the quality of life for patients and poses a significant burden on healthcare systems and socio-economic structures, particularly in the aftermath of the coronavirus disease 2019 (COVID-19) pandemic [3]. The pathological characteristics of knee osteoarthritis include joint cartilage damage, bone remodeling, osteophyte formation, synovial fluid accumulation, as well as abnormalities in the surrounding muscles and ligaments [4]. Although the exact pathogenic mechanisms of OA remain incompletely elucidated, factors such as genetics, age, gender, trauma, and metabolic disorders are considered essential contributing factors [5].

Insulin receptor substrate 2 (IRS2) is a key intermediary molecule in the insulin signaling pathway, playing a critical role in various biological processes such as cell survival, proliferation, and metabolism [6–8]. A reduction in IRS2 expression is strongly associated with insulin resistance and gene mutations[9]. Existing studies have shown that individuals with type 2 diabetes mellitus (T2DM) are more susceptible to osteoarthritis (OA) [10, 11]. Notably, both T2DM and OA are linked through complex mechanisms, but while the relationship between IRS2 and T2DM, as well as between T2DM and OA, has been well established, the potential role and expression changes of IRS2 in the pathogenesis of OA remain unclear.

Forkhead box protein O1 (FOXO1) is a transcription factor belonging to the FOXO family, with crucial regulatory functions in cellular processes such as survival, proliferation, differentiation, metabolism, and oxidative stress response. In the meniscus of OA patients, the expression levels of FOXO1 and FOXO3 are significantly reduced compared to normal meniscus, and the combined deficiency of FOXO1, 3, and 4 induces abnormal development in mouse meniscus by suppressing autophagy and the expression of antioxidant-related genes [8]. Recent studies have also revealed that FOXO1 maintains the homeostasis of joint chondrocytes by inducing the expression of genes related to synthesis, metabolism, and autophagy [9]. However, its potential molecular regulatory mechanisms are not yet fully understood.

Studies indicate that tyrosine-phosphorylated IRS proteins recruit and activate phosphatidylinositol 3-kinase (PI3K), subsequently promoting 3-phosphoinositide-dependent protein kinase 1 (PDK1)/AKT signal transduction, mediating the phosphorylation, activation, and nuclear exclusion of FOXO1 [12]. Additionally, research has revealed a significantly increased incidence of diabetes in Huntington’s (HD) patients. One probable reason is the recruitment of IRS2 into mutant Huntingtin protein (mHTT), hindering the activation of the PI3K/AKT/FOXO1 signaling pathway in pancreatic β-cells upon glucose stimulation [13]. These findings provide a potential framework for investigating whether IRS2 similarly regulates chondrocyte homeostasis through this mechanism.

Mitochondria, known as the “powerhouse of the cell”, execute various biochemical processes, playing a crucial role in oxidative phosphorylation and adenosine triphosphate (ATP) synthesis. Mitophagy, the selective removal of damaged mitochondria, is essential for regulating cellular function and maintaining cell homeostasis under stress conditions [14, 15]. Research suggests a close association between mitochondrial dysfunction and insulin resistance. Impaired mitophagy in chondrocytes often leads to extracellular matrix degradation and promotes OA progression [15, 16].

In this study, we identified IRS2 as the most significantly downregulated molecule in osteoarthritis through bioinformatics analysis. We hypothesize that IRS2 regulates mitochondrial autophagy via the PI3K/AKT pathway, which modulates FOXO1 activation. The downregulation of IRS2 impairs mitochondrial autophagy, leading to the accumulation of harmful oxidative stress and disrupting the metabolic homeostasis of chondrocytes, ultimately contributing to OA pathogenesis. Our findings suggest that exploring the role of IRS2 in osteoarthritis may provide valuable insights into its involvement in disease development and inform the design of therapeutic strategies.

## Methods

### Clinical samples collection and ethical approval

After obtaining approval from the Ethics Committee of Nanjing First Hospital, Nanjing Medical University (KY20230918-KS-01), we acquired twelve tibial plateaus from consenting osteoarthritis (OA) patients undergoing total knee replacement surgery. The medial portion of each knee tibial plateau was utilized for the damaged group, while the lateral tibial plateau with comparatively less cartilage damage served as the intact group. All experiments involving humans followed the *Declaration of Helsinki* [17]. Clinical trial number: not applicable.

### Analysis of the gene expression omnibus (GEO) database

We retrieved gene expression microarray datasets encompassing OA and normal human knee joint cartilage from the GEO public database and downloaded OA gene expression profiles GSE178557, GSE169077, GSE64394, and GSE57218. To integrate multiple datasets, we initially employed the R package inSilicoMerging for dataset merging [18]. Subsequently, we further applied the method proposed by Johnson WE et al. to remove batch effects, ultimately obtaining a batch-effect-corrected matrix [19].

The limma package was employed for the analysis of differentially expressed genes (DEGs) between patients and control groups, with filtering criteria set at *p* < 0.01 and |FC| >1.5 [20]. This facilitated the identification of DEGs in normal versus OA cartilage. The visualization of gene expression patterns between normal and OA groups was accomplished using the “ggplot2” and “pheatmap” R packages.

In this study, we employed the “glmnet” R package to perform regression analysis using the lasso-cox method. The regression analysis yielded five genes, with a Lambda value set at 0.183424729303045.

We utilized the CIBERSORT package in the R software to compute the proportions of 22 distinct immune cell types for patients exhibiting diverse immune profiles within the dataset [21]. Box plots were employed to visually represent the immune cell compositions among patients with varying immune patterns. The differences in immune cell proportions were assessed using the Wilcoxon rank-sum test, with a significance threshold set at *p* < 0.05. Statistical significance was considered when *p* < 0.05.

### Isolation and culture of primary articular chondrocytes

Primary mouse knee articular chondrocytes were meticulously isolated from mouse knee cartilage. The articular cartilage specimens were finely diced and subjected to a digestion process with 0.2% collagenase II (Biosharp, #BS164) at 37 °C for 10 h or more. Subsequently, the digested chondrocytes underwent filtration through 70-µm nylon filters (Biosharp, #BS-70-CS).

The cultured chondrocytes were nurtured in DMEM/F12 medium (Biosharp, #BL305A) supplemented with 10% fetal bovine serum (Excell, #FND500; v/v), 100 U/mL penicillin, and 100 µg/mL streptomycin (Beyotime, #C0222). The culture medium was diligently replaced every two days to maintain optimal conditions. Using collagen type II alpha 1 chain (COL2A1) immunofluorescence for phenotypic characterization of harvested primary mouse knee joint chondrocytes (Figure S2 A).

Cultures were maintained in an incubator at 37 °C with a humidified atmosphere containing 5% CO_2_. Upon reaching 70–80% confluency, tert-Butyl hydroperoxide (TBHP, Macklin, #B802372,) was introduced to induce reactive oxygen species (ROS) production.

The cells were subjected to infection with the designated multiplicity of infection (MOI) using either control adenovirus (Ad-C) or adenovirus expressing *Irs2* (Ad-*Irs2*, #ADV-262384). The adenoviruses for mouse *Foxo1* knockdown (Ad-m-*Foxo1*-shRNA, #shADV-259590) or the GFP shRNA silencing Adenovirus were procured from Vector Biolabs (Malvern, PA). A transfection efficiency of > 80% was considered successful and the cells were then used for subsequent experiments.

### Cell culture and maintenance

The ATDC5 cell line (Cat# 305427), derived from a mouse teratocarcinoma, was purchased from Cytion (Hamburg, Germany). This chondroprogenitor cell line was cultured and maintained in DMEM/F12 medium (Biosharp, #BL305A) supplemented with 10% fetal bovine serum (Excell, #FND500), 100 U/mL penicillin, and 100 µg/mL streptomycin (Beyotime, #C0222). Cells were maintained in a humidified incubator at 37 °C with 5% CO₂. The culture medium was replaced every two days.

ATDC5 cells were seeded for differentiation at a density of 6.0 × 10⁴ cells/cm² in multi-well plates. After 24 h, the maintenance medium was replaced, and to induce a uniform inflammatory state, all cells were pre-treated for 24 h with 10 ng/mL of recombinant mouse IL-1β (PeproTech, Cat# 211-11B). Following this pre-treatment, cells were washed with Phosphate-Buffered Saline (PBS) and subsequently cultured for 14 days in one of the four following experimental groups: (i) a vehicle control group, treated with differentiation medium containing 0.1% Dimethyl Sulfoxide (DMSO); (ii) an ITS-G induction group, treated with differentiation medium supplemented with 1X ITS-G Supplement (100X) [22](Thermo Fisher Scientific, Cat# 41400045); (iii) an Ad-Irs2 treatment group, where cells were transduced with an adenovirus expressing mouse Insulin Receptor Substrate 2 (Ad-Irs2) at a pre-determined Multiplicity of Infection (MOI); and (iv) an adenovirus control group, where cells were transduced with a corresponding empty adenovirus vector at the same MOI. The respective treatment media for all four groups were replaced every two to three days throughout the 14-day culture period.

### Alcian blue staining for cartilage matrix

The accumulation of sulfated glycosaminoglycans (sGAGs) in the extracellular matrix was assessed by Alcian Blue staining. Cells were washed twice with PBS and fixed with 4% paraformaldehyde for 20 min at room temperature. After fixation, the cells were rinsed with distilled water and stained for 30 min at room temperature using an Alcian Blue Staining Solution (pH 2.5) (Solarbio, Cat# G1560). Following staining, the solution was aspirated, and the plates were washed three times with distilled water to remove excess stain. The stained plates were air-dried, and images of the cartilage nodules were captured using a light microscope.

### Reverse transcription-quantitative polymerase chain reaction (RT-qPCR) of mRNA

RNA was extracted from the cells utilizing the TRIzol reagent (ThermoFisher, #15596018CN). Subsequently, a first-strand cDNA was synthesized from 0.5 µg of total RNA, employing the SweScript RT II First Strand cDNA Synthesis Kit (With gDNA Remover, Servicebio, #G3333-50). The real-time reverse transcriptase RT-qPCR reaction was executed using the ABI QuantStudio 5 system. To assess gene expression, the RT-qPCR data were quantified relative to the housekeeping gene *Gapdh*.

### Cell viability assay

For the cell viability assessment, a cell suspension (100 µL/well) was seeded into a 96-well plate, and the culture plate was incubated in a CO_2_ incubator for 24 h. Subsequently, the designated treatments were applied and incubated for either 24–48 h. Following the treatment, 10 µL of CCK-8 solution (Beyotime, #C0037) was introduced to each well. The culture plates were returned to the incubator and incubated for an additional 2 h. The optical density at 450 nm was quantified using a Sunrise microplate reader (TECAN, Männedorf, Switzerland).

### Antibodies

Anti-IRS2 (ab134101, Abcam), Anti-MMP13 (18165-1-AP, Proteintech), Anti-ACAN (AHP0022, Invitrogen), Anti-GAPDH (60004-1-Ig, Proteintech), Anti-COL2A1 (sc-52658, Santa Cruz), Anti-SOX9 (ab185966, Abcam), Anti-Beclin-1 (A21191, Abclonal), Anti-ATG7 (10088-2-AP, Proteintech), Anti-LC3 (#2775S, Cell Signaling Technology), Anti-AKT (60203-2-Ig, 1: 5000, Proteintech), Anti-Phospho-AKT (#4060S, Cell Signaling Technology), Anti-FOXO1 (#2880, Cell Signaling Technology), Anti-Phospho-FOXO1 (RK05760, Abclonal), Anti-Histone-H3 (17168-1-AP, Proteintech), Anti-Mouse secondary antibody (BL001A, Biosharp), Anti-Rabbit secondary antibody (BL003A, Biosharp).

### Western blot analysis

For tissue samples, after cutting into fine pieces with scissors, add lysate (Beyotime, #P0013B) containing 1mM PMSF (Beyotime, #ST506). Homogenize with a homogenizer until fully lysed. For cell samples, the method is as described previously [23]. Briefly, after removing the culture medium, add an appropriate amount of lysis solution directly, and lyse on ice for 5 min. Subsequent steps follow the guidelines provided by the reagent manufacturer. Proteins were separated through 10% sodium dodecyl sulfate-polyacrylamide gel electrophoresis (SDS-PAGE) (Biosharp, #BL565A), and then transferred to polyvinylidene fluoride (PVDF) membranes (Millipore, #IPVH07850). After blocking with 5% skim milk for an hour, the membrane was incubated with primary antibodies overnight at 4 °C. After washing with Tris-buffered saline with 0.1% Tween 20 detergent (TBS-T) for 3 × 10 min, incubated at 37 °C for an hour with secondary antibodies. Immunoreactive proteins were visualized by enhanced chemiluminescence and light detection by Tanon 5200 (Tanon, China). Relative quantitative analysis was performed using ImageJ 1.54 F.

### Mitochondria analysis

Mitochondrial transmembrane potential and mitochondrial morphology were assessed using MitoTracker Red CMXRos (Beyotime, #C1049B). the location of lysosomes using LysoTracker Green was visualized using LysoTracker Green (Beyotime, #C1047S). Mouse chondrocytes were stained with 50 nM MitoTracker Red and 50 nM LysoTracker Green for 30 min at 37 °C and nuclei were stained with Hoechst 33,258 (Beyotime, # C1011) for 10 min. Microscopic observation was performed with a fluorescence microscope (Zeiss, Germany), and fluorescence intensity was measured using ImageJ 1.54 F.

### Mouse OA model and intra-articular injection

The destabilization of the medial meniscus (DMM) was performed on 12-week-old male C57BL/6 mice (purchased from Nanjing First Hospital) to induce osteoarthritis (OA). Knee joint tissues were harvested four weeks post-surgery [24]. Ethical approval for animal-based studies was obtained from the Ethics Committee of Nanjing First Hospital, Nanjing Medical University (DWSY-2101052). All animal experiments strictly adhered to the guidelines outlined in the National Institutes of Health’s Guide for the Care and Use of Laboratory Animals. The mice were housed in specific pathogen-free conditions with a 12-hour light and 12-hour dark cycle at a temperature of approximately 22 ~ 23 °C.

For the OA model, animals were anesthetized with 75 mg/kg ketamine and 10 mg/kg xylazine before undergoing unilateral DMM procedures. The surgical protocol was meticulously performed in accordance with the methodology outlined by S.S. Glasson B.V.Sc [24].

To elucidate the involvement of IRS2 in OA, mice subjected to either sham or DMM surgery received intra-articular injections of 1 × 10^9^ plaque-forming units (pfu) of control adenovirus (Ad-C) or adenovirus expressing murine *Irs2* (Ad-*Irs2*).

All laboratory mice were disposed in accordance with the American Veterinary Medical Association (AVMA) Guidelines for the Euthanasia of Laboratory Animals (2020).

### Measurement of intracellular ROS by flow cytometry

Chondrocytes seeded in six-well plates were treated with IL-1β and Ad-Irs2 for 24 h, followed by incubation with 10 µM 2′,7′-dichlorofluorescein diacetate (DCFH-DA) at 37 °C for 20 min to assess intracellular ROS levels. We measured the fluorescence of dichlorofluorescein (DCF), the oxidized product of DCFH-DA, using an excitation wavelength of 488 nm and an emission wavelength of 525 nm. After staining, the cells were analyzed using flow cytometry (Thermo Fisher Scientific), and the resulting fluorescence intensity was quantified using FlowJo software version 10.7 (BD Life Sciences, Franklin Lakes, NJ, USA).

### Micro-CT analysis

Micro-CT analysis was conducted to specifically evaluate structural changes in subchondral bone, such as osteophyte formation and subchondral bone sclerosis, which are critical pathological features in OA progression. Knee joints were extracted, fixed in 4% paraformaldehyde for 24 h, and scanned using SkyScan1176 (Bruker, Germany). The manufacturer’s software was used for 3D reconstruction and analysis.

### Histological and immunohistochemical analyses

Four weeks post-DMM, mouse long bones were harvested with the knee joint preserved and fixed in 10% neutral buffered formalin for 24 h at room temperature. Following fixation, the specimens underwent decalcification in 10% EDTA (pH 7.4) for 4 weeks at room temperature with gentle agitation, with the EDTA solution changed every 3–4 days to ensure effective decalcification[25]. Subsequent processing, including paraffin embedding, and sectioning, was conducted by ServiceBio in Wuhan, China. The specimens underwent Haematoxylin and Eosin staining, immunohistochemical staining, and Safranin O/Fast green staining (Sigma Aldrich, Germany), following the instructions provided by the respective reagent manufacturers.

For immunohistochemical staining, the Super Plus™ High Sensitive and Rapid Immunohistochemical Kit (Elabscience, #E-IR-R220) was utilized for subsequent procedures. Briefly, antigen retrieval coincided with heat restoration, followed by peroxidase inactivation. The primary antibody was incubated overnight at 4 ℃, and the corresponding secondary antibody was applied at room temperature for 30 min. After DAB color development, haematoxylin staining was performed, followed by dehydration, transparency, and mounting.

In tissue immunofluorescence, knee joint sections were deparaffinized, washed, and blocked with 10% goat serum for 1 h at room temperature. Primary antibodies were applied in 0.1% Tween 20 and incubated overnight at 4 °C, followed by secondary antibody using Alexa Fluor488, 568, 647-conjugated secondary antibody (Abcam, #ab150077, #ab175473, #ab15011) for 1 h at room temperature, and stained with DAPI for 3–5 min.

In cellular immunofluorescence, mouse chondrocytes were rinsed with Phosphate-Buffered Saline (PBS), fixed in 4% paraformaldehyde, and permeabilized in 0.1% Triton X-100 for 15 min. Subsequently, cells were blocked with 5% bovine serum albumin for 30 min at 37 °C, washed with PBS, and incubated overnight at 4 °C. Following washing, cells were incubated with Alexa Fluor488-conjugated secondary antibody (Abcam, #ab150077, #ab175473, #ab150115) for 1 h at room temperature and stained with DAPI for 3–5 min. Fluorescence signals were detected and captured using Axio Imager.A2 (Zeiss, Germany).

### Statistical analysis

Quantitative analysis of Western blots, immunofluorescence, and immunohistochemistry was performed using ImageJ software (National Institutes of Health, USA). All data are presented as mean ± standard deviation (mean ± S.D). Statistical comparisons between the two groups were conducted using an unpaired, two-tailed Student’s t-test. For comparisons among three groups, a one-way analysis of variance (ANOVA) followed by Dunnett’s multiple comparison tests was applied. For all statistical analyses, differences with *p* values < 0.05 were considered statistically significant, and experiments were repeated for indicted times in the figure legend. All statistical analyses were performed using GraphPad Prism software version 9.5 (GraphPad Software, Inc.).

## Results

### Screening of differentially expressed genes (DEGs) and Kyoto Encyclopedia of Genes and Genomes (KEGG) pathway enrichment analysis

GSE178557, GSE169077, GSE64394, and GSE57218 were obtained from Gene Expression Omnibus (GEO). These samples were from healthy populations and osteoarthritis patients. We utilized the R package inSilicoMerging to merge the datasets. As is shown in the density graphic and box plots, the sample distributions of the datasets were quite different (Figure S1A, B, and C). The empirical Bayes method was used to adjust the batch effects of the merged datasets and the results indicated that the batch effect was removed effectively.

**Fig. 1:**
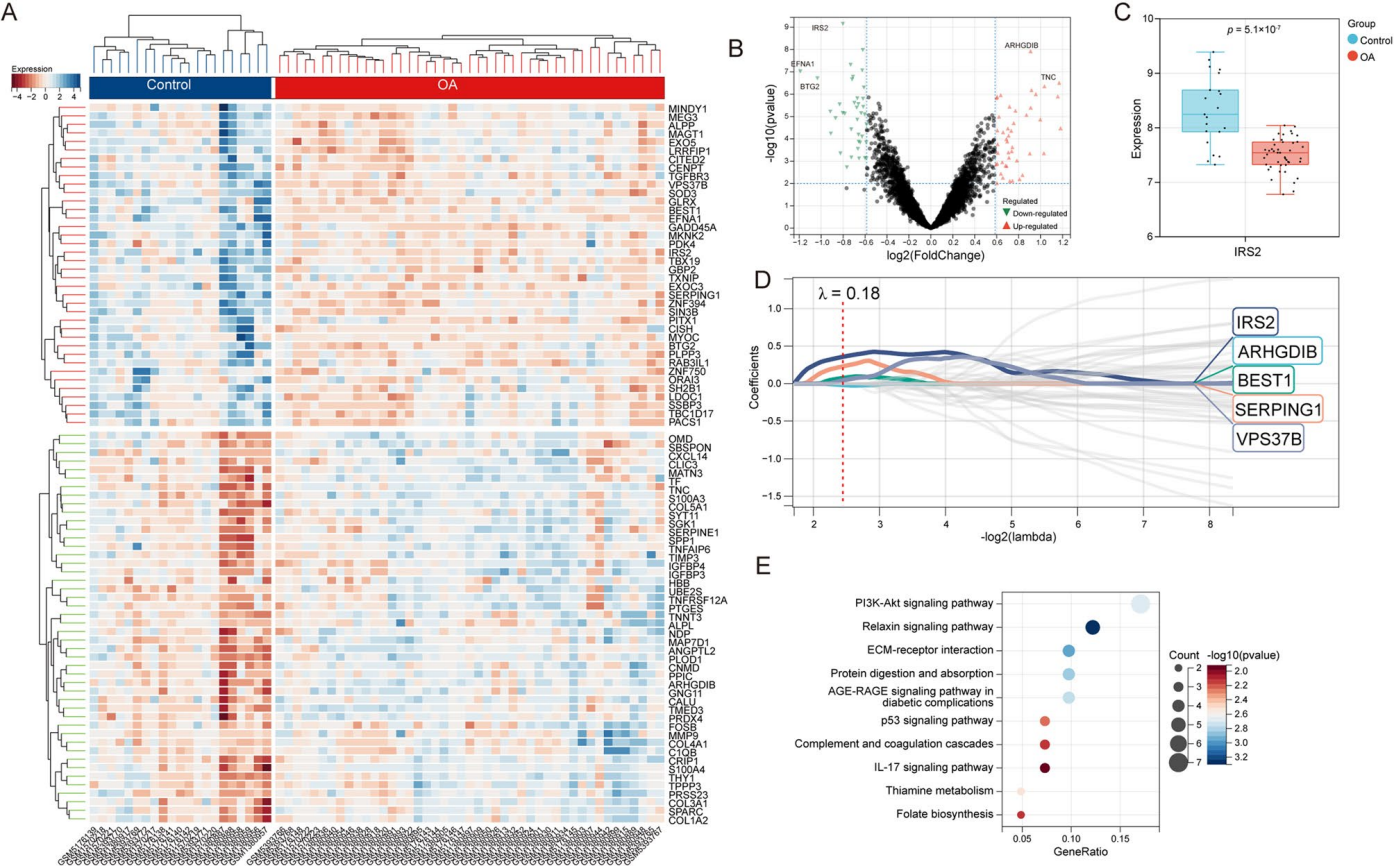
Screening of differentially expressed genes (DEGs) and Kyoto Encyclopedia of Genes and Genomes (KEGG) pathway enrichment analysis.** A** Heatmap of DEGs. **B** Volcano plot of DEGs. **C** The expression level of *IRS2* in the merged dataset. **D** Least absolute shrinkage and selection operator (LASSO) regression analysis was used to select the genes. **E** KEGG pathway enrichment analysis of the differentially expressed gene

After the merging and standardization of the datasets, 86 DEGs (up = 46, down = 40) were identified using the “limma” package (Table S2, Fig. 1A, B). Among these DEGs, *IRS2* exhibits the most significant differential expression, ranking first (*p* = 5.1 × 10^−7^) (Fig. 1C). The LASSO regression analysis yielded 5 genes (*IRS2*, *ARHGDIB*, *BEST1*, *SERPING1* and *VPS37B*) from the optimal model (Fig. 1D, S1D, E). Therefore, we hypothesized that IRS2 may be a key target in the OA process. Furthermore, KEGG pathway enrichment analysis of the differentially expressed genes was performed (Fig. 1E), revealing a significant enrichment of the PI3K-AKT signaling pathway.

### The expression of IRS2 is reduced in osteoarthritic joints and IL-1β-stimulated chondrocytes

**Fig. 2:**
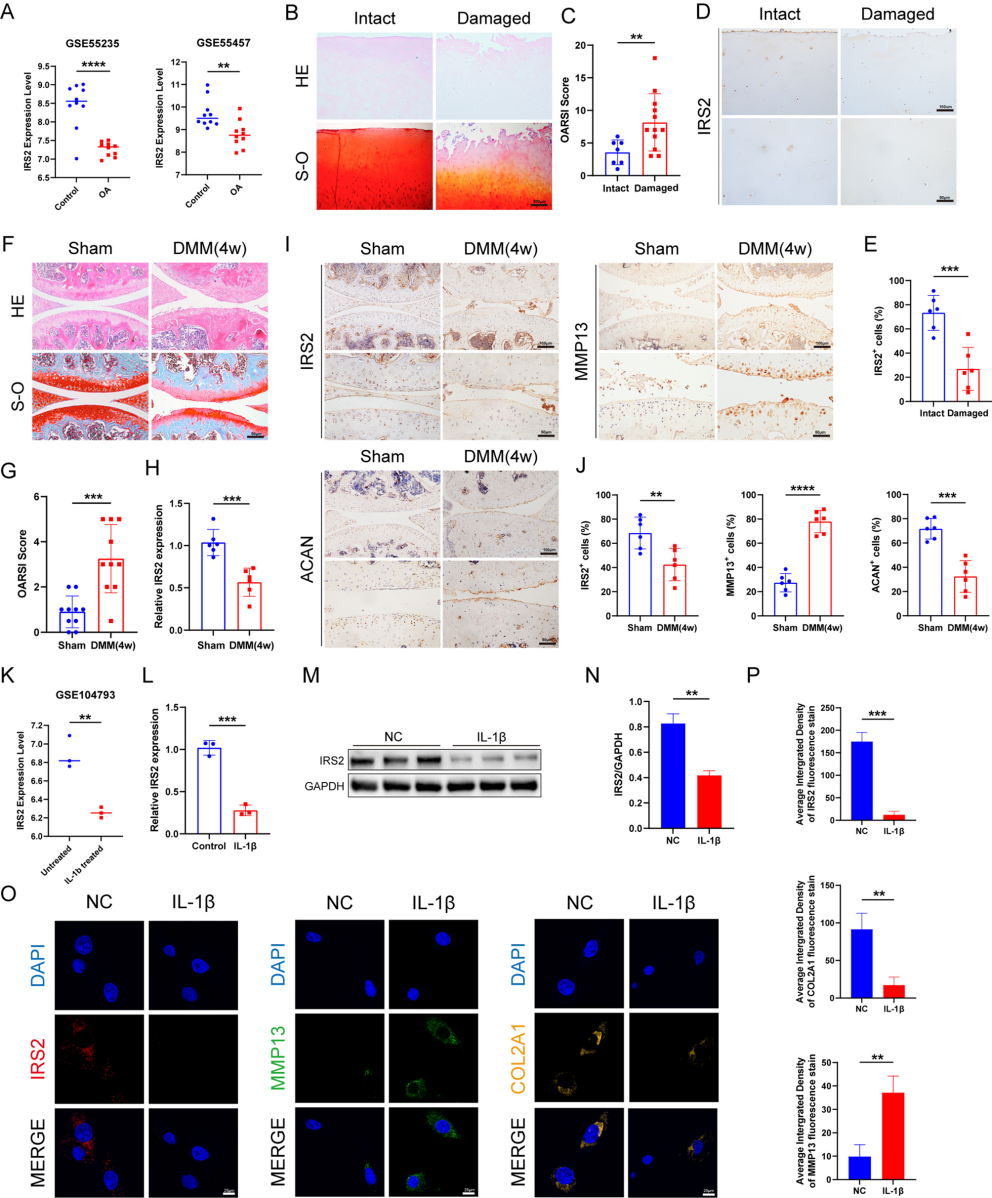
The expression of IRS2 is reduced in osteoarthritic joints and IL-1β-stimulated chondrocytes. **A** Boxplot of IRS2 expression in healthy samples and OA samples from GSE55235 and GSE55457. **B** Representative images of HE and Safranin O-stained sections of human knee joints. **C** OARSI score of human knee joints. **D**,** E **Representative images and quantification of immunohistochemical staining of IRS2 of human knee articular cartilage. **F** mRNA expression level of *IRS2* in mouse chondrocytes. **G** Representative images of HE and Safranin O-stained sections of mouse knee joints. **H** OARSI score of knee joints in two groups of mice. **I**,** J** Representative images and quantification of immunohistochemical staining and quantification of IRS2, MMP13 and ACAN of mouse knee articular cartilage. **K**,** L** IRS2 expression level in untreated and IL-1β treated primary mouse articular chondrocytes. **M**,** N** Western blot analysis and quantification of IRS2 in untreated and IL-1β treated mouse chondrocytes. **O**,** P** Representative images and quantification of Immunofluorescence staining of IRS2, MMP13 and COL2A1 in untreated and IL-1β treated mouse chondrocytes. Data are presented as mean ± S.D. For panel C, n = 7 (Intact) and n = 12 (Damaged). For panel E, n = 10 per group. For mouse samples (H, J), n = 8 per group. For in vitro experiments (L, N, P), n = 3 per group. Statistical significance was determined by a two-tailed Student’s t-test, with p-values shown on each graph

In datasets using human-derived samples, we verified that IRS2 expression was significantly reduced in OA articular cartilage (Fig. 2A). We subsequently performed histological staining on the tibial plateau cartilage obtained from individuals undergoing human knee arthroplasty, revealing a significant reduction in the protein level of IRS2 in OA (Fig. 2B-E). We employed a unilateral destabilization of the medial meniscus (DMM) to establish a murine knee osteoarthritis (OA) model (Fig. 2F, G) and, through qPCR analysis, observed a significant decrease in IRS2 mRNA levels in the articular cartilage of DMM-induced OA knee joints (Fig. 2H). After histological staining of mouse knee joint sections, we further confirmed a significant reduction in IRS2 protein levels in DMM-induced osteoarthritis (OA), accompanied by an increase in matrix metalloproteinases 13 (MMP13) and a decrease in Aggrecan (ACAN) (Fig. 2I, J). To further elucidate the role of IRS2 in chondrocytes, we initially validated the decreased expression of IRS2 in primary mouse chondrocytes subjected to IL-1β intervention using the GSE104793 dataset. Subsequently, we employed in vitro stimulation of mouse chondrocytes with 10 ng/ml of IL-1β, confirming our findings at both the transcriptional and protein levels. (Fig. 2K-N). The results of immunofluorescence demonstrated a significant increase in MMP13 expression and a significant decrease in collagen type II alpha 1 chain (COL2A1) expression in mouse chondrocytes under IL-1β stimulation. This was concomitant with a marked reduction in IRS2 expression (Fig. 2O, P). The results of these in vivo and in vitro experiments further confirm the potential key role played by IRS2 in OA.

### The overexpression of IRS2 restores chondrocyte dysfunction induced by IL-1β

**Fig. 3:**
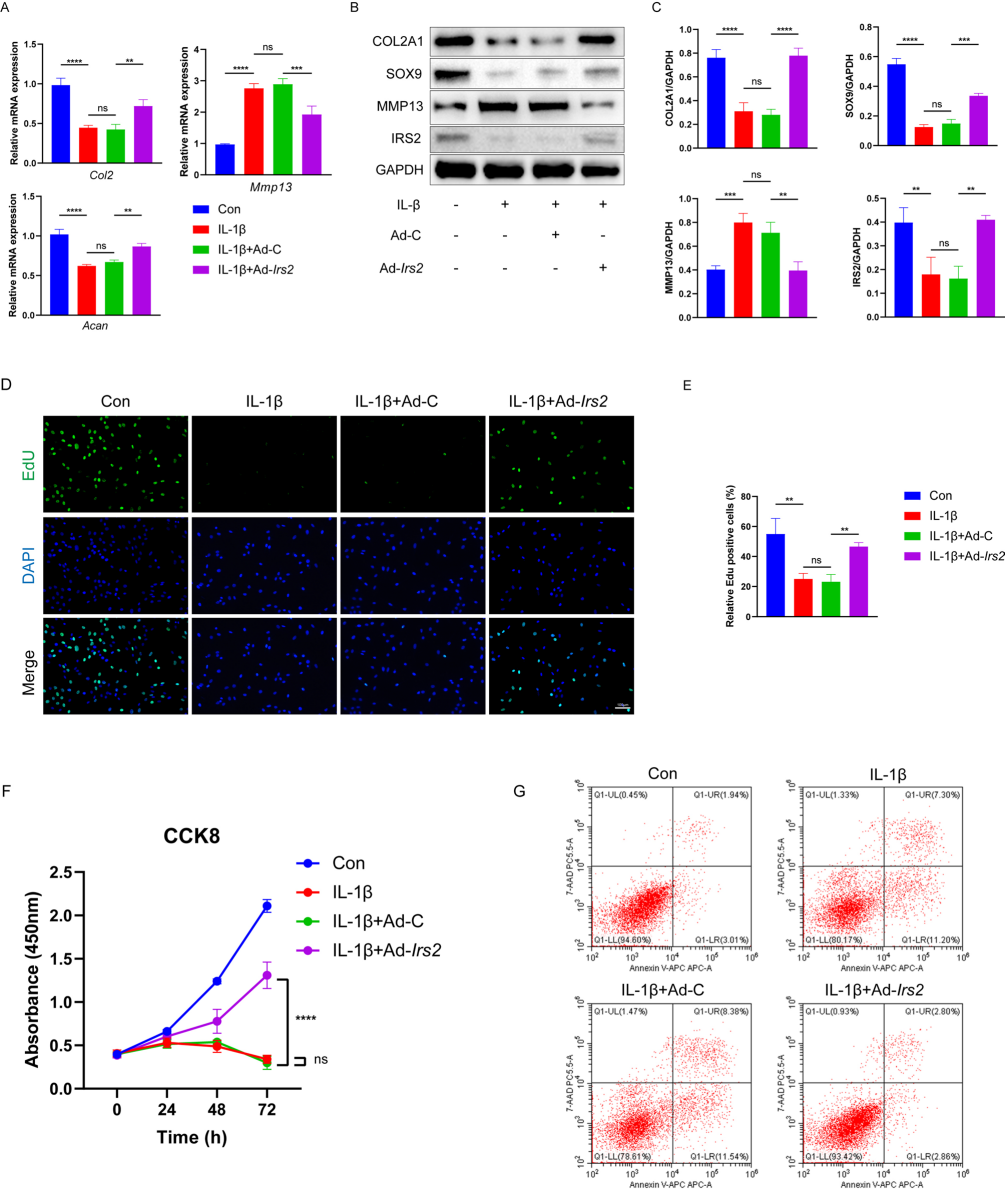
The overexpression of IRS2 restores chondrocyte dysfunction induced by IL-1β. **A** Relative mRNA expression levels of *Col2a1*, *Mmp13* and *Acan* in four groups of mouse chondrocytes. **B**,** C **Western blot analysis and quantification of COL2A1, SOX9, MMP13 and IRS2 in four groups of mouse chondrocytes. **D**,** E** EDU staining and quantification of four groups of primary chondrocytes (EDU-positive chondrocyte, green; DAPI, blue). **F** The proliferation ability of four groups of mouse chondrocytes analyzed by CCK8 assay. **G** Apoptosis of chondrocytes among four groups by flow cytometry. Data in A, C, E, and F are presented as mean ± S.D. (n = 3 per group). Statistical significance was determined by one-way ANOVA followed by Dunnett’s multiple comparisons test. Significance is indicated by the p-value on each graph

To further validate the role of IRS2 in chondrocytes, we utilized adenovirus transfection to overexpress IRS2 in primary mouse chondrocytes. qPCR results revealed that the elevated levels of *Mmp13* and the decreased levels of the extracellular *Acan* and *Col2a1* induced by IL-1β stimulation were alleviated in mouse chondrocytes overexpressing IRS2 (Fig. 3A). The Western blot results were consistent with the qPCR findings, demonstrating that the expression levels of cartilage functional differentiation and extracellular matrix synthesis proteins, including sry-box transcription factor 9 (SOX9), could be restored with the overexpression of IRS2. Conversely, the extracellular matrix degradation protein MMP13 was reduced due to IRS2 overexpression. These findings confirmed that IRS2 overexpression could restore the functional loss of chondrocytes induced by IL-1β (Fig. 3B, C). Markedly increased EdU-positive chondrocytes evidenced that IRS2 overexpression could restore the proliferative capacity of chondrocytes (Fig. 3D, E). Additionally, the proliferation capacity of chondrocytes was assessed using CCK-8, and the results were consistent with the EdU findings. The overexpression of IRS2 reversed the reduced proliferative ability of chondrocytes induced by IL-1β (Fig. 3F). According to the Annexin V/PI method, the cell apoptosis rate of the control group, IL-1β group, IL-1β + Ad-C group, and IL-1β + Ad*-Irs2* group was observed. After IL-1β treatment for 24 h, the chondrocyte apoptosis rate in the IL-1β group and IL-1β + Ad-C group was significantly increased compared with the control group. The chondrocyte apoptosis rate in the IL-1β + Ad*-Irs2* group was significantly lower than that in the IL-1β + Ad-C group (Fig. 3G).

### The overexpression of IRS2 can restore autophagy in OA chondrocytes

**Fig. 4:**
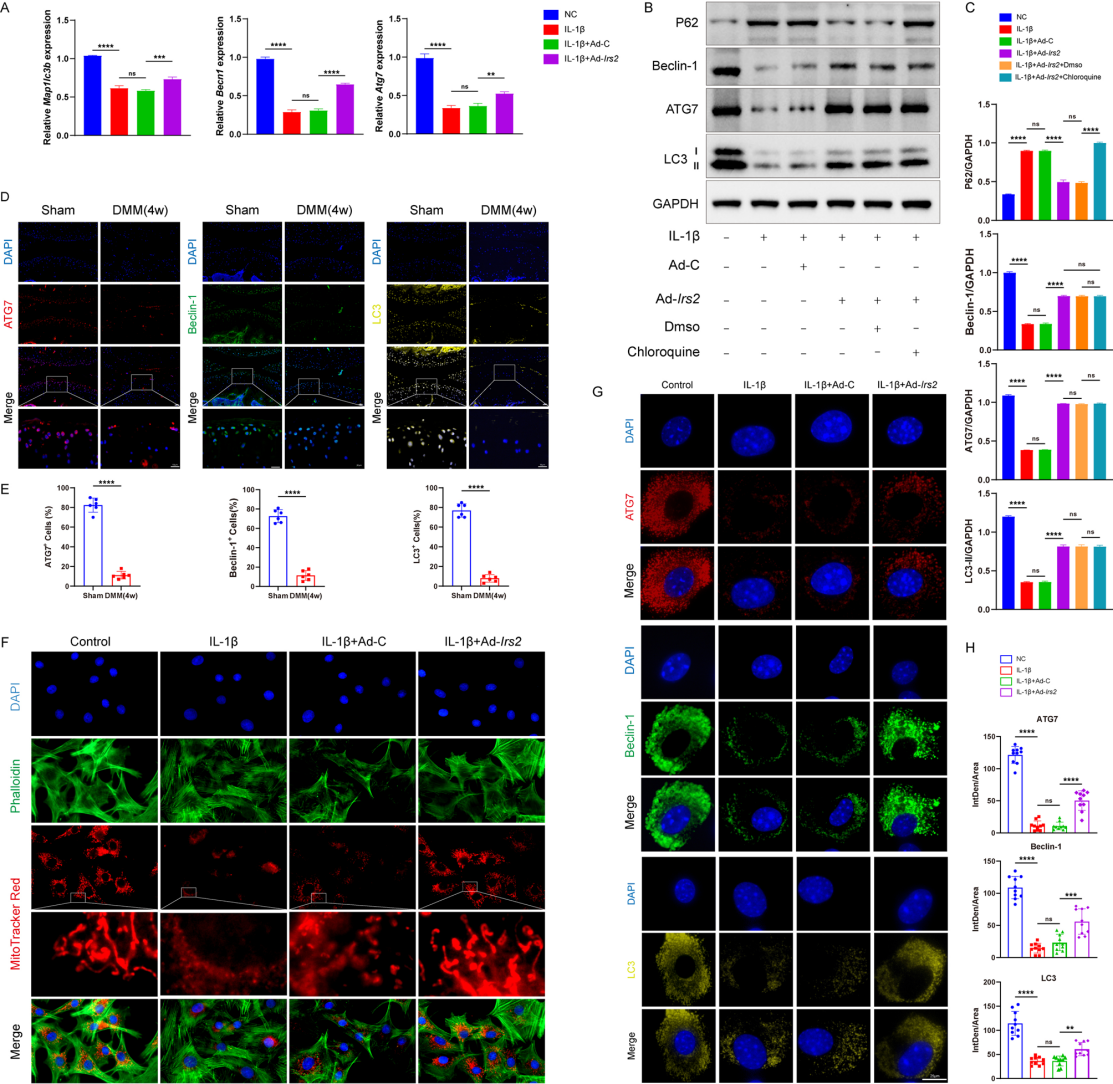
The overexpression of IRS2 can restore autophagy in OA chondrocytes. **A **Quantification of *Map1lc3b*, *Atg7* and *Becn1* mRNA expression levels in four groups of mouse chondrocytes. **B**,** C** Western blot analysis and quantification of P62, Beclin-1, ATG7 and LC3 in six groups of mouse chondrocytes. **D**,** E** Representative images and quantification of immunofluorescence staining of ATG7, Beclin-1 and LC3 in Sham and DMM groups of mouse knee articular cartilage. **F **Representative images of MitoTracker Red staining in four groups of mouse chondrocytes. **G**,** H** Representative images of immunofluorescence staining (**G**) and corresponding quantification (**H**) of ATG7, Beclin-1 and LC3 in four groups of mouse chondrocytes. Data are presented as mean ± S.D. For in vivo immunofluorescence quantification (E), n = 10 per group and significance was determined by a two-tailed Student’s t-test. For all in vitro experiments (**A, C,** and **H**), n = 3 per group. Statistical significance was determined by a two-tailed Student’s t-test for two-group comparisons (**D**) or by one-way ANOVA with Dunnett’s multiple comparisons test for multi-group comparisons (**A, C, H**). P-values are shown on each graph

Adequate autophagic levels play a crucial role in maintaining the physiological homeostasis of chondrocytes [15]. We utilized RT-qPCR technology to analyze the impact of overexpressed IRS2 on the activity of the autophagy pathway in chondrocytes under interleukin treatment. The results revealed that following IL-1β treatment, the transcription levels of autophagy initiation protein Beclin-1, as well as autophagy-related 7 (ATG7), an E1-like enzyme and microtubule-associated protein 1 light chain 3 II (LC3-II), were significantly reduced. However, upon overexpression of IRS2, the transcription levels of these autophagy-related genes showed a partial recovery (Fig. 4A). Western blot analysis corroborated these findings at the protein level. Treatment with IL-1β decreased the expression of Beclin-1, ATG7, and the conversion to LC3-II, while causing an accumulation of the autophagy substrate P62, indicating a blockage of autophagic flux. Notably, the addition of chloroquine, an autophagy inhibitor that blocks lysosomal degradation, led to a greater accumulation of LC3-II in IRS2-overexpressing cells compared to the IL-1β-treated group, confirming that IRS2 restores the autophagic flux rather than just inducing autophagosome formation. Overexpression of IRS2 successfully reversed these effects, increasing Beclin-1, ATG7, and LC3-II levels while reducing P62 accumulation (Fig. 4B, C). In a destabilization of the medial meniscus (DMM)-induced mouse osteoarthritis model, tissue immunofluorescence technique similarly indicated a decrease in chondrocyte autophagic activity in OA, including ATG7, Beclin-1, and LC3 (Fig. 4D, E). To further confirm these findings at a cellular level, we performed immunofluorescence on cultured chondrocytes. The results showed that IL-1β treatment diminished the formation of distinct fluorescent puncta representing ATG7, Beclin-1, and LC3. In contrast, overexpression of IRS2 significantly restored the formation of these autophagosome-associated puncta, indicating a recovery of autophagosome generation (Fig. 4G, H). Mitochondria are strongly associated with the normal development and maintenance of cartilage. Chondrocytes can regulate the morphology and function of mitochondria through autophagy. Using MitoTracker staining, we observed that IL-1β promoted the fragmentation and fission of chondrocyte mitochondria, along with a corresponding decrease in mitochondrial membrane potential. However, overexpression of IRS2 partially restored a more filamentous mitochondrial morphology and rescued the membrane potential (Fig. 4F).

### IRS2 regulates chondrocyte autophagy activity and oxidative stress levels through the phosphorylation of FOXO1

**Fig. 5:**
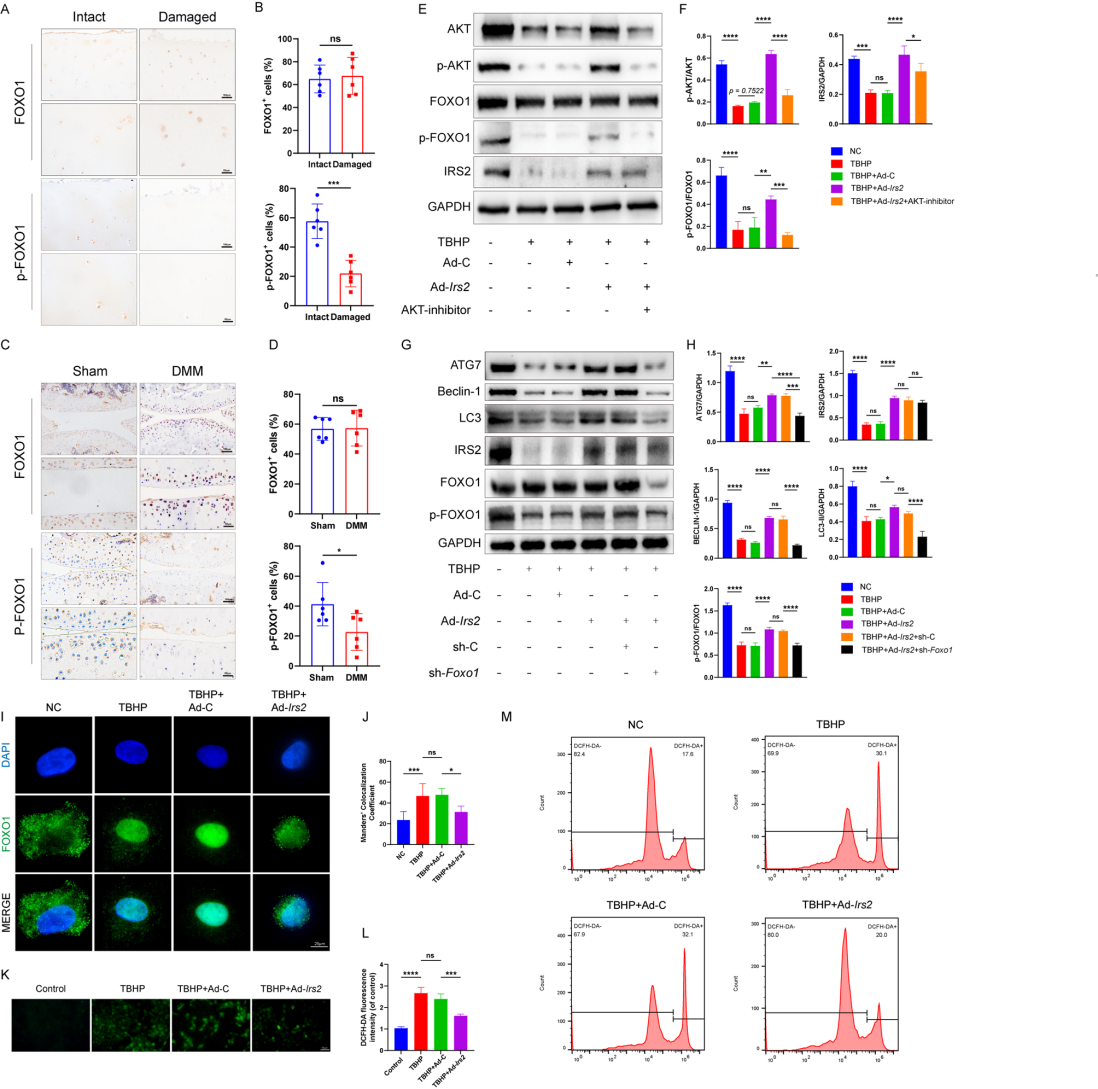
IRS2 regulates chondrocyte autophagy activity and oxidative stress levels through the phosphorylation of FOXO1. **A** Representative immunohistochemistry image showing FOXO1 and p-FOXO1 protein expression in human knee joint sections. Brown staining indicates positive FOXO1 expression in specific tissue regions. **B** Quantification of FOXO1 and p-FOXO1 protein expression in Intact and Damaged groups of human knee cartilage. **C** Representative immunohistochemistry image showing FOXO1 and p-FOXO1 protein expression in mouse knee joint sections. **D** Quantification of FOXO1 and p-FOXO1 protein expression in Sham and DMM groups of mouse knee joints. **E** Western Blot images illustrate the detection of AKT, p-AKT, FOXO1, p-FOXO1 and IRS2. Experimental conditions include treatments with TBHP, TBHP + Ad-C, TBHP + Ad*-Irs2*, TBHP + AKT-inhibitor and a control group. **F** Western Blot results were quantified, and the bar graph depicts the relative intensity of protein expression in diverse groups. Each bar represents the mean, and error bars indicate standard error. **G** Western Blot images illustrate the detection of ATG7, Beclin-1, LC3, IRS2, FOXO1 and p-FOXO1. Experimental conditions include treatments with TBHP, TBHP + Ad-C, TBHP + Ad*-Irs2*, TBHP + sh-C, TBHP + sh-*Foxo1* and a control group. **H** Western Blot results were quantified, and the bar graph depicts the relative intensity of protein expression in diverse groups. Each bar represents the mean, and error bars indicate standard error. **I** Representative images of immunofluorescence staining from NC, TBHP, TBHP + Ad-C, and TBHP + Ad*-Irs2*. FOXO1 is visualized in green, and DAPI staining (blue) marks the cell nuclei. **J** Quantification of FOXO1 nuclear localization was performed using Manders’ colocalization coefficient, providing a relative measure of FOXO1 protein co-localization with DAPI. **K** Representative images of chondrocytes stained with DCFH-DA for ROS detection, depicting chondrocytes from four experimental groups. **L** Bar chart illustrating the relative intensity of DCFH-DA fluorescence in each group. **M** Measurement of ROS levels using DCFH-DA fluorescent probe in four groups. Data are presented as mean ± S.D. For human IHC quantification (**B**), n = 6 per group. For mouse IHC quantification (**D**), n = 6 per group. For all in vitro experiments (**F, H, J, L, M**), n = 3 per group. Statistical significance was determined by a two-tailed Student’s t-test for two-group comparisons (**B, D**) or by one-way ANOVA with Dunnett’s multiple comparisons test for multi-group comparisons (**F, H, J, L**). P-values are shown on each graph.

FOXO1 is strongly associated with the autophagic flux in chondrocytes. Therefore, we hypothesized that IRS2 regulates autophagy by modulating the expression of FOXO1. In both human and destabilization of the medial meniscus (DMM)-induced mouse osteoarthritis models, we observed that the protein levels of FOXO1 did not show significant changes compared to normal cartilage, while the phosphorylation levels of FOXO1 were significantly reduced in OA cartilage (Fig. 5A-D). Oxidative stress-related apoptosis and autophagy play crucial roles in the development of OA, a progressive cartilage degenerative disease with multifactorial etiologies. Therefore, we further validated the relationship between FOXO1 and IRS2 in mouse primary chondrocytes cultured in vitro. Western blot results demonstrated that the protein levels of p-FOXO1 significantly recovered in chondrocytes after overexpression of IRS2 compared to TBHP treatment. Additionally, to rule out other possible post-translational modifications of FOXO1 (such as acetylation), we used an AKT inhibitor to suppress FOXO1 phosphorylation without affecting other post-translational modifications. The results showed that when chondrocytes were treated with the AKT inhibitor, the protein levels of FOXO1 did not change significantly, and overexpression of IRS2 did not increase the phosphorylation levels of FOXO1 (Fig. 5E, F). To investigate whether FOXO1 was a key protein regulated by IRS2 in chondrocyte autophagy, we knocked down the expression of FOXO1 using sh-*Foxo1*. Western blot results showed that after FOXO1 knockdown, overexpression of IRS2 could not restore the autophagic activity of chondrocytes (Fig. 5G, H).

Immunofluorescence results showed that TBHP stimulation caused FOXO1 to translocate into the cell nucleus, and overexpression of IRS2 could reduce TBHP-induced nuclear translocation of FOXO1 (Fig. 5I, J). These results further confirmed that FOXO1 is an intermediate protein through which IRS2 regulated chondrocytes autophagy. We used DCFH-DA for chondrocyte reactive oxygen species (ROS) staining, and the results showed that overexpression of IRS2 could reduce the IL-1β-induced increase in ROS (Fig. 5K-M). These findings suggested that IRS2 played a regulatory role in chondrocyte autophagy and oxidative stress through the phosphorylation of FOXO1.

### p-FOXO1 regulates chondrocyte mitophagy through interaction with ATG7

**Fig. 6:**
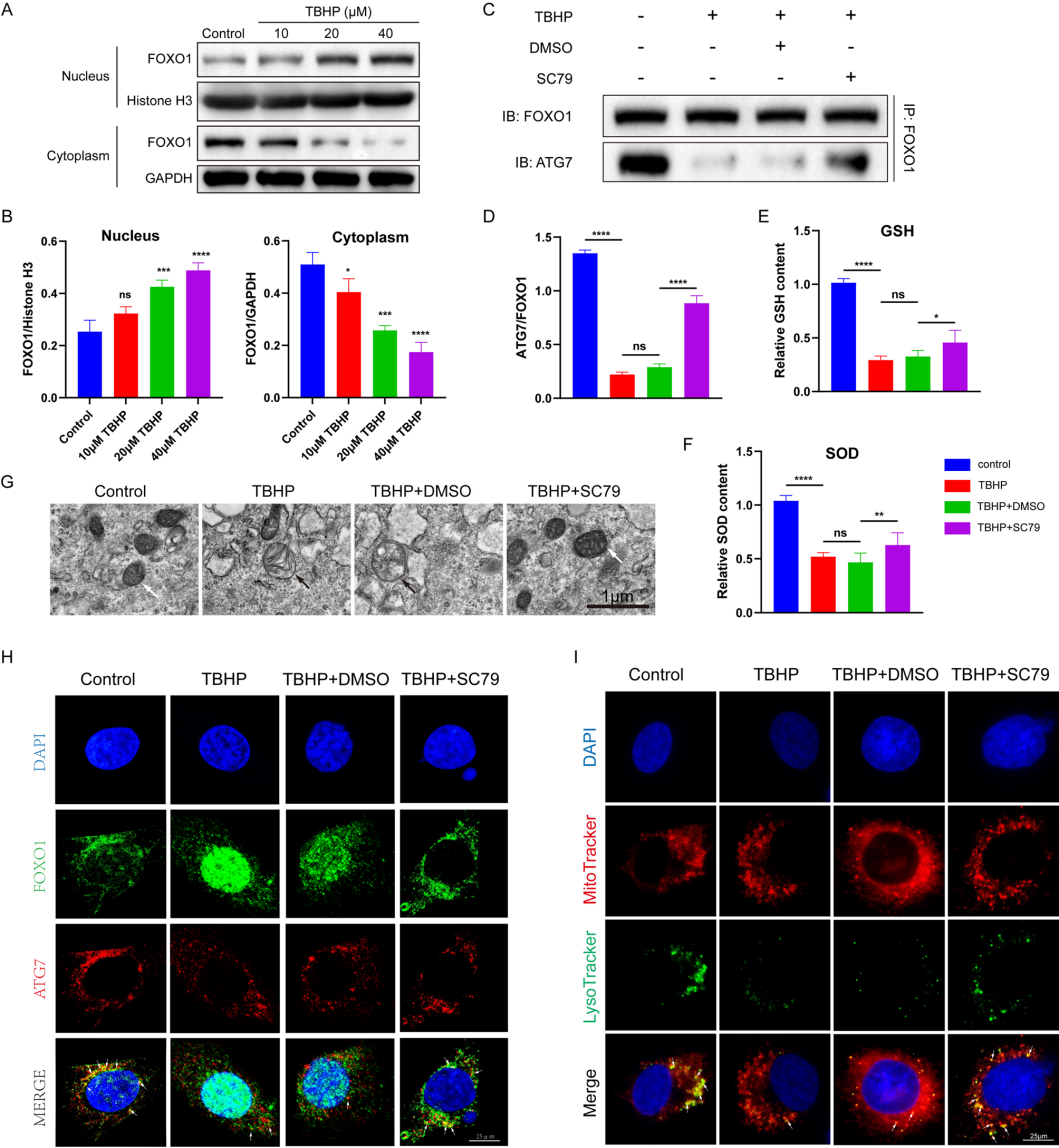
p-FOXO1 regulates chondrocyte mitophagy through interaction with ATG7. **A** Representative Western blot bands showing the expression levels of nuclear and cytoplasmic FOXO1 in chondrocytes treated with escalating concentrations of TBHP (10, 20 40µM). **B** Quantification of Western blot results depicting the relative expression of nuclear and cytoplasmic FOXO1. Data are presented as mean ± standard deviation. **C** Western blot analysis of ATG7 co-immunoprecipitated with FOXO1 in chondrocytes. **D** Quantification of p-FOXO1 and ATG7 Interaction in chondrocytes. **E** Relative quantification of GSH levels in four groups, each bar represents the mean ± standard deviation. **F** Relative quantification of SOD levels in four groups, each bar represents the mean ± standard deviation. **G** Transmission Electron Microscopy (TEM) images depicting mitochondrial morphology in chondrocytes from four experimental groups. White arrows: normal mitochondrial morphology. Black arrows: abnormal Mitochondria morphology. **H** Co-localization of FOXO1 and ATG7 in Chondrocytes Revealed by Immunofluorescence Double Staining. FOXO1: green; ATG7: red. **I** Representative images of chondrocytes subjected to fluorescence double staining. Mitochondria are labeled in red (MitoTracker Red), while lysosomes are labeled in green (LysoTracker Green). Data in B, D, E, and F are presented as mean ± S.D (n = 3 per group). Statistical significance was determined by one-way ANOVA followed by Dunnett’s multiple comparisons test. P-values are shown on each graph

We initially stimulated chondrocytes with a gradient of TBHP for 24 h and observed that with increasing TBHP concentrations, FOXO1 gradually translocated into the nucleus, consistent with previous research findings (Fig. 6A, B) [26]. Literature has reported phosphorylated cytosolic FoxO1 associates with Atg7 to induce autophagy in iNKs[27, 28]. Thus, we hypothesized that cytosolic p-FOXO1 could facilitate chondrocyte autophagy by binding to ATG7. Immunoprecipitation results showed that TBHP treatment reduced the interaction between FOXO1 and ATG7, in line with previous results, partially explaining the downregulation of the chondrocyte autophagy pathway induced by TBHP. However, after treatment with the AKT activator SC79, the interaction level between FOXO1 and ATG7 significantly increased. Since activated AKT leads to the phosphorylation and nuclear export of FOXO1, this suggested that p-FOXO1, not Ac-FOXO1, participated in the autophagy regulation induced by ATG7 in chondrocytes (Fig. 6C, D).

Levels of the antioxidant substances GSH and SOD were measured in chondrocytes cultured in vitro, revealing that SC79 significantly increased intracellular GSH and SOD levels (Fig. 6E, F). Transmission electron microscopy was used to observe the mitochondrial morphology of chondrocytes, showing that TBHP stimulation caused mitochondrial swelling and fragmentation, while SC79 improved mitochondrial morphology (Fig. 6G). Immunofluorescence staining further supported our hypothesis: after treatment with SC79, FOXO1 translocated out of the nucleus, and the interaction between p-FOXO1 and ATG7 was significantly restored (Fig. 6H). Staining of chondrocyte mitochondria and lysosomes using MitoTracker Red and LysoTracker Green, respectively, similarly indicated that SC79 treatment restored mitochondrial autophagy in chondrocytes (Fig. 6I).

### Intra-articular injection of adenovirus expressing Irs2 improves DMM-induced knee OA in mice

**Fig. 7:**
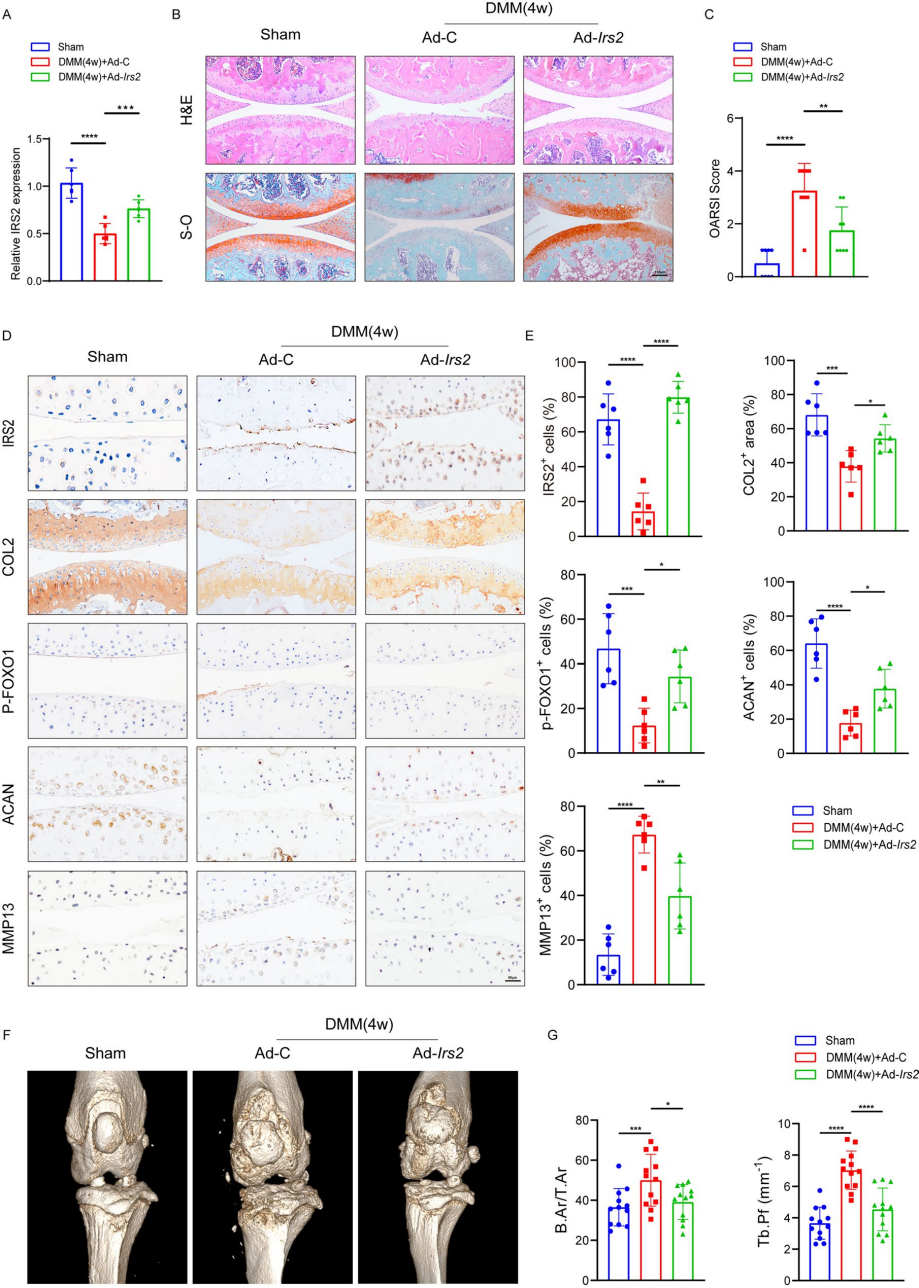
Intra-articular injection of r adenovirus expressing ***Irs2*** improves DMM-induced knee OA in mice. **A** Expression level of IRS2 mRNA in the knee articular cartilage of sham, DMM(4w) + Ad-C, and DMM(4w) + Ad-*Irs2* mice. **B** Hematoxylin and Eosin (H&E) staining and Safranin O-Fast Green staining of cartilage and extracellular matrix components in knee joint sections from Sham, DMM + Ad-C, and DMM + Ad-Irs2 groups. **D** Immunohistochemical analysis of IRS2, p-FOXO1, COL2, ACAN, and MMP13 in the knee joint sections. **E** Relative quantification of IRS2, p-FOXO1, COL2, ACAN, and MMP13 protein expression in the knee joint sections. **F** Representative micro-CT images illustrating the overall structure of mouse knee joints in three experimental groups. **G** Bar chart presenting the Bone Area to Tissue Area ratio (B.Ar/T.Ar) and Trabecular Pattern Factor (Tb.pf) in three experimental groups. The data is presented as mean ± standard deviation. Data are presented as mean ± S.D. The number of biological replicates (n) per group was as follows: n = 6 for qPCR analysis (**A**), n = 6 for OARSI scoring (**C**), n = 6 for immunohistochemistry quantification (**E**), and n = 12 for micro-CT analysis (**G**). Statistical significance was determined by a Kruskal-Wallis H test for Ordinal categorical variable (**C**) or by one-way ANOVA with Dunnett’s multiple comparisons test for multi-group comparisons (**A, E, G**). P-values are shown on each graph

To delve into the functional role of IRS2 in mouse knee joint cartilage, we employed a strategy of intra-articular injection of adenovirus expressing *Irs2*. PCR validation revealed that intra-articular injection of adenovirus overexpressing *Irs2* in DMM mice significantly increased Irs2 expression in chondrocytes (Fig. 7A). Hematoxylin and eosin (HE) staining results demonstrated an improvement in the cartilage damage on the surface of the mouse knee joint injected with Ad-*Irs2*. Safranin O/Fast Green staining similarly indicated that Ad-*Irs2* injection could restore the loss of sulfated matrix and surface irregularities induced by destabilization of the medial meniscus (DMM) (Fig. 7B, C). Immunohistochemistry results revealed that Ad-*Irs2* injection increased the content of p-FOXO1 protein in the knee joint cartilage layer. Additionally, the protein expression levels of IRS2, COL2A1 and ACAN were partially restored, while MMP13 was reduced compared to the DMM group (Fig. 7D, E).

Results from micro-CT indicated that Ad-*Irs2* improved the formation of osteophytes and subchondral bone sclerosis caused by DMM, as well as the decreased smoothness of the joint surface (Fig. 7F). Calculated parameters such as Bone Area to Tissue Area ratio (B.Ar/T.Ar) and Trabecular Pattern Factor (Tb.pf) based on micro-CT results suggested that Ad-*Irs2* ameliorated the changes in knee joint osteoproliferation, trabecular thickening, and trabecular structure induced by DMM (Fig. 7G). Additionally, the hot plate and knee extension tests showed that intra-articular of Ad-*Irs2* significantly alleviates OA-induced knee pain in mice (Figure S4). These findings suggest that intra-articular injection of Ad-*Irs2* significantly ameliorates DMM-induced knee arthritis in mice.

## Discussion

**Fig. 8:**
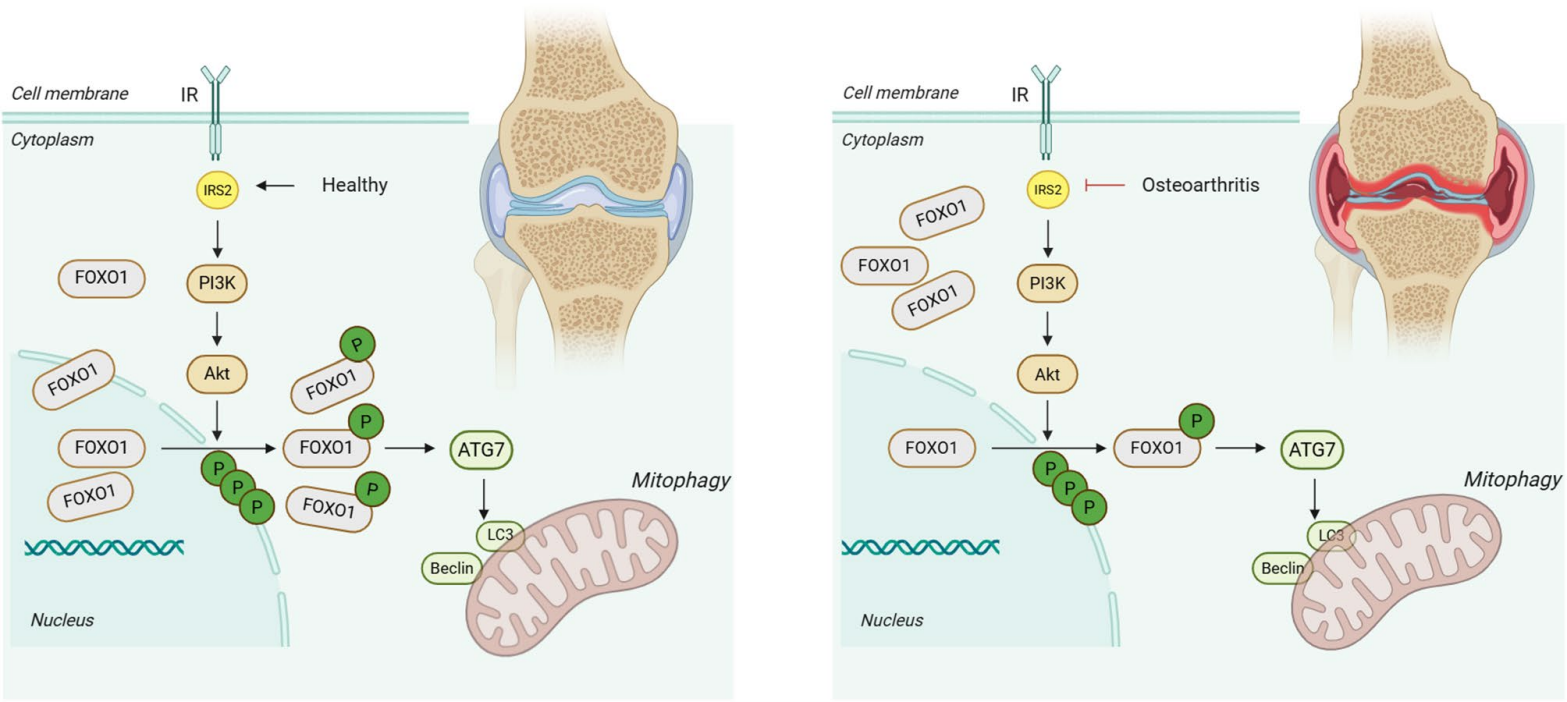
The role of IRS2/FOXO1 signaling in chondrocyte autophagy and osteoarthritis progression. In normal chondrocytes, IRS2 activation stimulates the downstream PI3K/AKT signaling pathway. Activated AKT phosphorylates FOXO1, leading to its activation and nuclear translocation. Phosphorylated FOXO1 interacts with ATG7 in the cytoplasm to initiate autophagy, thereby maintaining cellular homeostasis (Left). Conversely, in osteoarthritis (OA) chondrocytes, the IRS2/FOXO1 pathway is suppressed. Although this change did not affect the total amount of FOXO1 in chondrocytes, the proportion of activated FOXO1 was downregulated, impairing autophagy. This disruption prevents the clearance of harmful metabolites, exacerbating cartilage degradation and OA progression (Right)

In this study, we focused on the role of IRS2 in chondrocytes and its involvement in osteoarthritis (OA). Using bioinformatics methods, we identified a significant downregulation of IRS2 expression in OA patients, suggesting that IRS2 may play a critical role in the pathophysiology of OA. We characterized the transcriptional and protein levels of IRS2 in human OA tissues, a mouse model of OA induced by destabilization of the medial meniscus (DMM), and IL-1β-stimulated chondrocytes, revealing a marked decrease in IRS2 expression. Subsequent experiments demonstrated that the effects of IRS2 downregulation could be alleviated via the PI3K/AKT pathway, which subsequently influences FOXO1 phosphorylation and regulates chondrocyte homeostasis (Fig. 8).

IRS2, an insulin receptor substrate protein, binds to class IA phosphatidylinositol 3-kinase (PI3K) upon phosphorylation of its tyrosine residues, leading to the production of phosphatidylinositol [3–5]-triphosphate (PIP3). PIP3 activates phosphoinositide-dependent kinase-1 (PDK1), which in turn activates a series of kinases, including AKT [29]. IRS2 is known to play a pivotal role in glucose homeostasis[30, 31]. While hyperglycemia and type 2 diabetes mellitus (T2DM) are believed to promote OA progression through oxidative stress and chronic low-grade inflammation, the relationship between glucose metabolism and OA remains a subject of ongoing debate [32–34].

Previous studies have emphasized the importance of IRS2 in obesity and metabolic regulation [35]. Our findings offer new insights into this area, particularly shedding light on the regulatory mechanism of FOXO1 phosphorylation through the PI3K/AKT pathway. Disruption of this signaling pathway in chondrocytes may lead to imbalances in extracellular matrix metabolism, potentially initiating the onset of OA. These results suggest that the molecular basis of IRS2 signaling transduction in various tissues may share a common foundation for metabolic regulation in chondrocytes.

The mammalian FOXO transcription factor family—comprising FOXO1, FOXO3, FOXO4, and FOXO6—regulates key biological processes such as development and aging[36]. FOXO1 is predominantly expressed in bone and cartilage tissues, where it maintains cartilage homeostasis. Studies have indicated that the TGF-β/TAK1-FOXO1 signaling axis plays a crucial role in regulating chondrocyte autophagy and homeostasis [26]. During meniscus homeostasis in OA, FOXO1 contributes to meniscus development and maturation by modulating autophagy and antioxidant defense mechanisms [37]. However, recent studies have suggested that FOXO1 may also exert a protective effect in chondrocytes, with its activation potentially mitigating OA progression. For example, FOXO1-activating compounds have been shown to reverse OA progression in some contexts [38]. Given the association between autophagic homeostasis and degenerative diseases [15], our study also focuses on the role of FOXO1 in cellular autophagy. Some studies suggest that phosphorylated FOXO1 in iNK cells regulates autophagy activity in the cytoplasm by interacting with ATG7 [27]. In contrast, other literature proposes that acetylated, rather than phosphorylated, FOXO1 plays a key role in autophagy regulation by interacting with ATG7 in the HCT116 colon cancer cell line [39]. Interestingly, our study found that phosphorylated FOXO1 regulates mitochondrial autophagy in chondrocyte cytoplasm through a non-acetylation-dependent pathway, thereby maintaining cellular homeostasis. This finding is crucial for understanding the mechanisms underlying metabolic imbalance in chondrocytes during OA. Moreover, other study have indicated that various post-translational modifications of FOXO1, including glycosylation and methylation, may also be involved in diverse physiological and pathological processes[40]. These different forms of post-translational modifications may interact with one another, providing important direction for our future research.

Despite these insights, we observed that the phosphorylation of FOXO1 in response to IRS2/PI3K/AKT signaling leads to FOXO1’s exclusion from the nucleus, inhibiting its transcriptional activity. This finding contrasts with the protective role attributed to FOXO1 in some reports. It suggests that the functional outcome of FOXO1 activation in OA may be context-dependent and influenced by various signaling pathways. Therefore, further research is necessary to clarify the dual roles of FOXO1 in OA and to explore the regulatory mechanisms that determine whether FOXO1 activation or inhibition is more beneficial in specific pathological conditions.

In our study, we demonstrated that AKT activation regulates FOXO1 by phosphorylation, controlling its nuclear exclusion and inhibiting its transcriptional function. This aligns with existing literature on the regulation of FOXO1 by AKT. In the context of glucose metabolism, AKT mediates insulin responses by phosphorylating FOXO1, thereby regulating energy homeostasis. In chondrocytes, the PI3K/AKT/FOXO1 pathway may play a similar role in the onset of OA, thus providing a molecular basis for the connection between metabolic disorders and joint diseases.

Despite the extensive analysis of IRS2/FOXO1 signaling in OA, certain limitations restrict the scope of our conclusions. Although we attempted to validate p-FOXO1 independent of FOXO1 acetylation using the AKT activator SC79, more direct and high-level evidence is needed to confirm that the interaction between p-FOXO1 and ATG7 occurs independently of other post-translational modifications. Additionally, while diabetes is believed to exacerbate bone and joint tissue damage, thereby promoting OA [41]. IRS2 mediates multifaceted signals triggered by insulin and other cytokine receptors, contributing to the progression of T2DM [42]. Constructing T2DM-induced OA models is essential to directly confirm the pivotal role of IRS2 in linking these two conditions. Furthermore, our current study has several temporal limitations that warrant consideration. Our in vivo study utilized a 4-week post-DMM model. While this timeframe is well-established for observing significant early OA-like cartilage changes and assessing the initial efficacy of interventions [24], we acknowledge that it only captures an early stage of this chronic disease. Future longitudinal studies employing longer endpoints, such as 8 and 12 weeks, are necessary to evaluate the long-term protective effects of IRS2 and its impact on late-stage disease progression. Similarly, our in vitro experiments focused on the acute, short-term effects of IRS2 overexpression. This approach was crucial for establishing the direct molecular link between IRS2 and the autophagy pathway in a controlled setting. However, it does not address the sustained efficacy of this intervention over time, which is critical in the context of a chronic, degenerative disease like OA. Therefore, future long-term studies are warranted to determine if the therapeutic effects regulated by IRS2 are durable.

## Conclusions

This study provides valuable insights into the role of the IRS2/AKT/FOXO1 axis in osteoarthritis (OA), uncovering a mechanism by which FOXO1 is regulated through autophagy in chondrocytes, independent of acetylation. These findings establish a link between this regulatory pathway and the onset of OA, offering a novel framework for understanding how metabolic and joint diseases are interconnected. Our results provide a foundation for developing future therapeutic strategies targeting this axis in OA.

## Supplementary Information

Supplementary Material 1.

Supplementary Material 2.

Supplementary Material 3.

Supplementary Material 4.

## Data Availability

No datasets were generated or analysed during the current study.

## Abbreviations

OA: Osteoarthritis
IRS2: Insulin Receptor Substrate 2
PI3K: Phosphoinositide 3-Kinase
AKT: Protein Kinase B
FOXO: Forkhead Box O
ATG7: Autophagy Related 7
DMM: Destabilization of the Medial Meniscus
COVID-19: Coronavirus Disease 2019
T2DM: Type 2 Diabetes Mellitus
ATP: Adenosine Triphosphate
PDK1: Phosphoinositide-Dependent Kinase-1
HD: Huntington's Disease
mHTT: Mutant Huntingtin
GEO: Gene Expression Omnibus
DEG: Differentially Expressed Genes
TBHP: Tert-Butyl Hydroperoxide
ROS: Reactive Oxygen Species
MOI: Multiplicity of Infection
MMP13: Matrix Metalloproteinase 13
ACAN: Aggrecan
GAPDH: Glyceraldehyde 3-Phosphate Dehydrogenase
COL2A1: Collagen Type II Alpha 1 Chain
SOX9: SRY-Box Transcription Factor 9
ATG7: Autophagy Related 7
LC3: Microtubule-associated Protein 1A/1B-light Chain 3
ANOVA: Analysis of Variance
B.Ar/T.Ar: Bone Area to Tissue Area ratio
Tb.pf: Trabecular Pattern Factor
TGF-β: Transforming Growth Factor-beta
