# Supplementary figures and images for: IRS2/FOXO1 mitigates osteoarthritis by regulating chondrocyte autophagy and mitochondrial function
Source: Mol Med. 2025 Sep 26;31:293. doi: 10.1186/s10020-025-01346-8 (PMC12465773; doi:10.1186/s10020-025-01346-8)

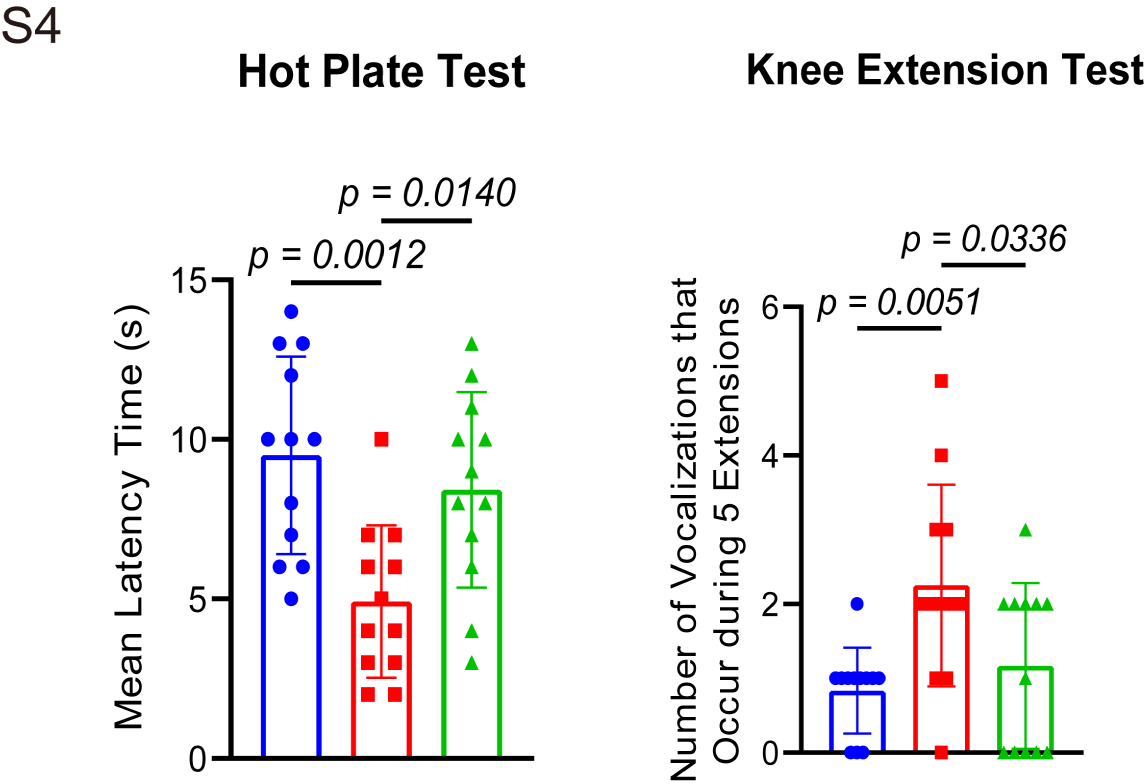

Supplement: Supplementary file 1 — Supplementary Material 1. [file 10020_2025_1346_MOESM1_ESM.tif]

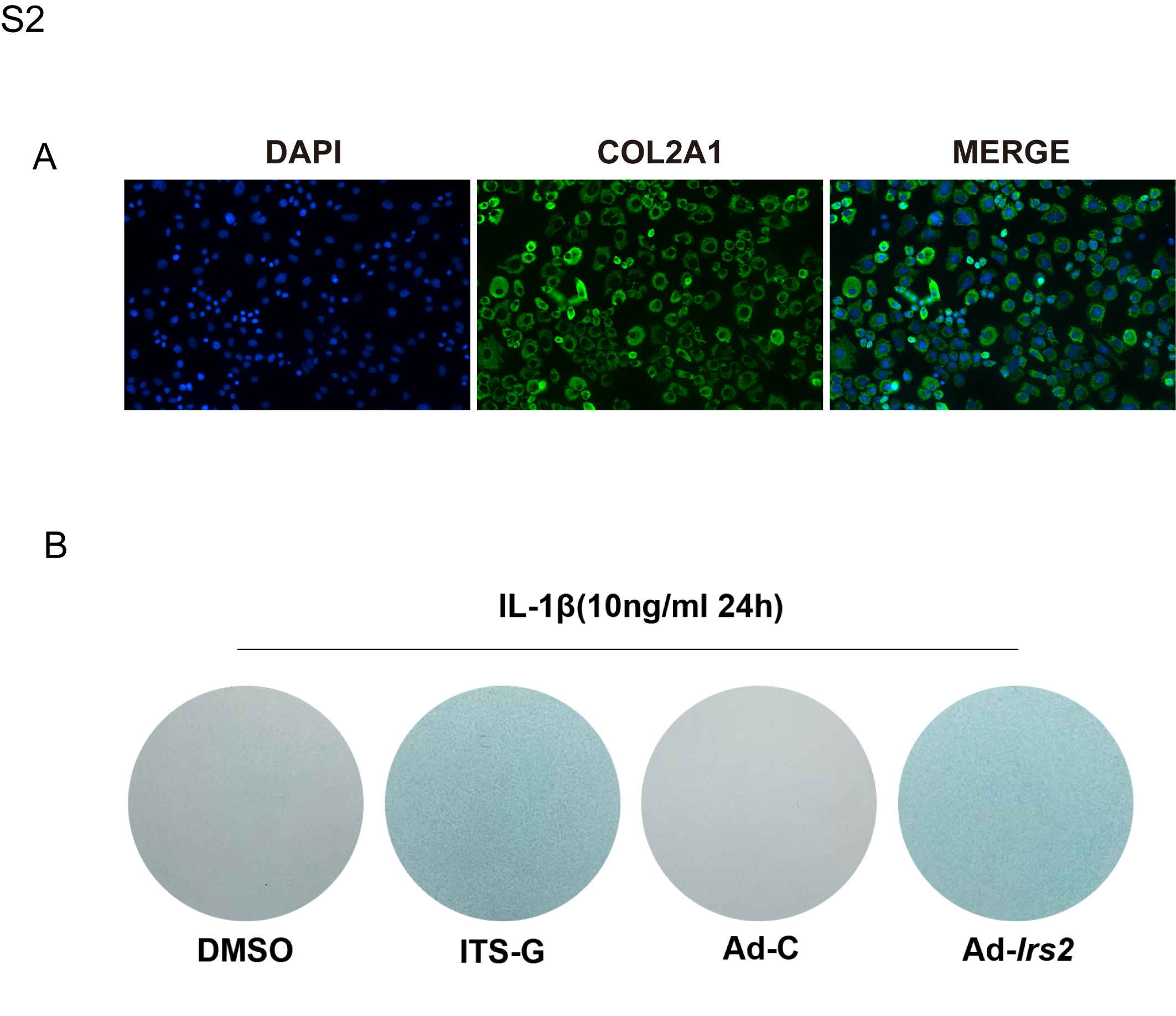

Supplement: Supplementary file 2 — Supplementary Material 2. [file 10020_2025_1346_MOESM2_ESM.jpg]

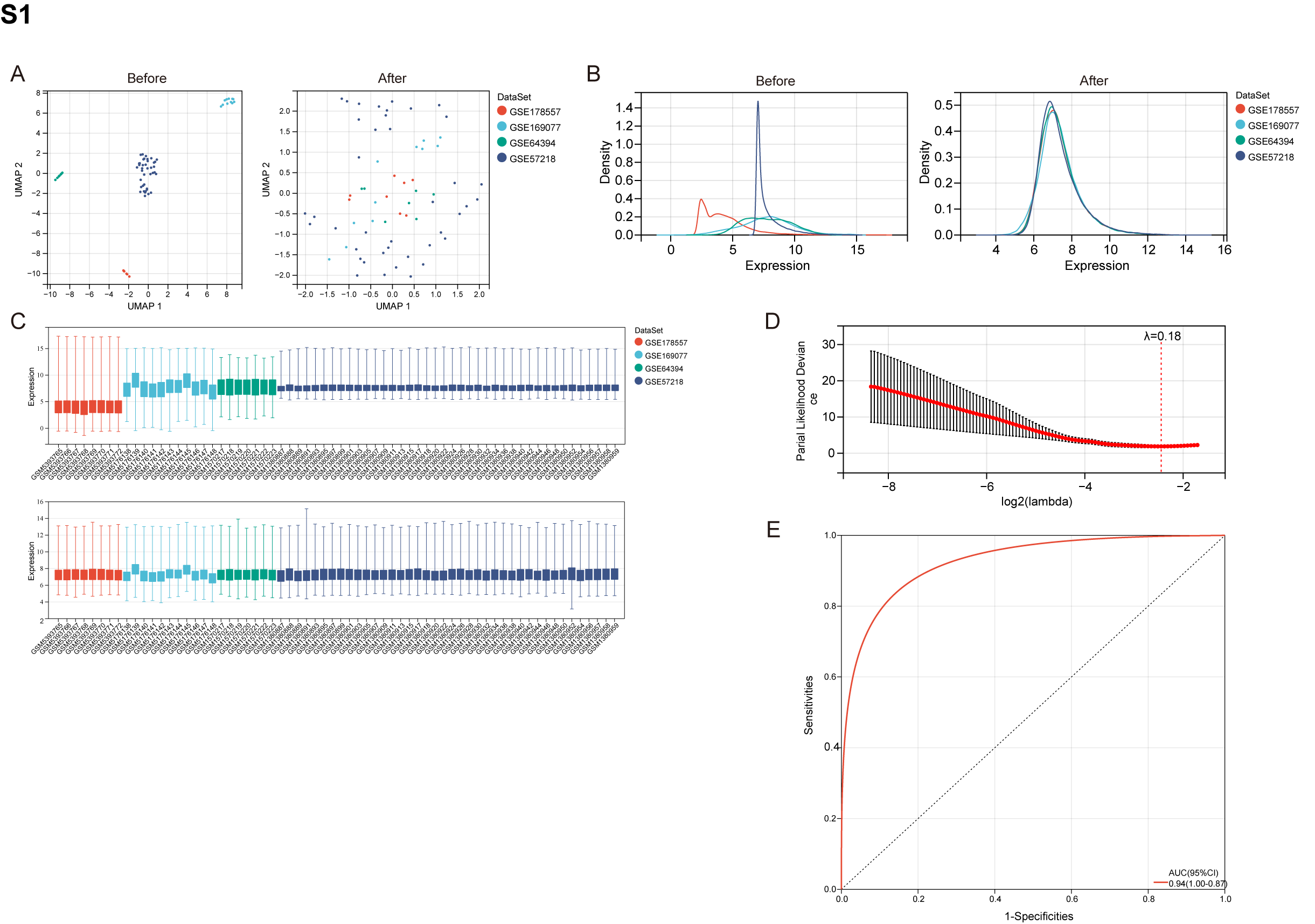

Supplement: Supplementary file 3 — Supplementary Material 3. [file 10020_2025_1346_MOESM3_ESM.tif]

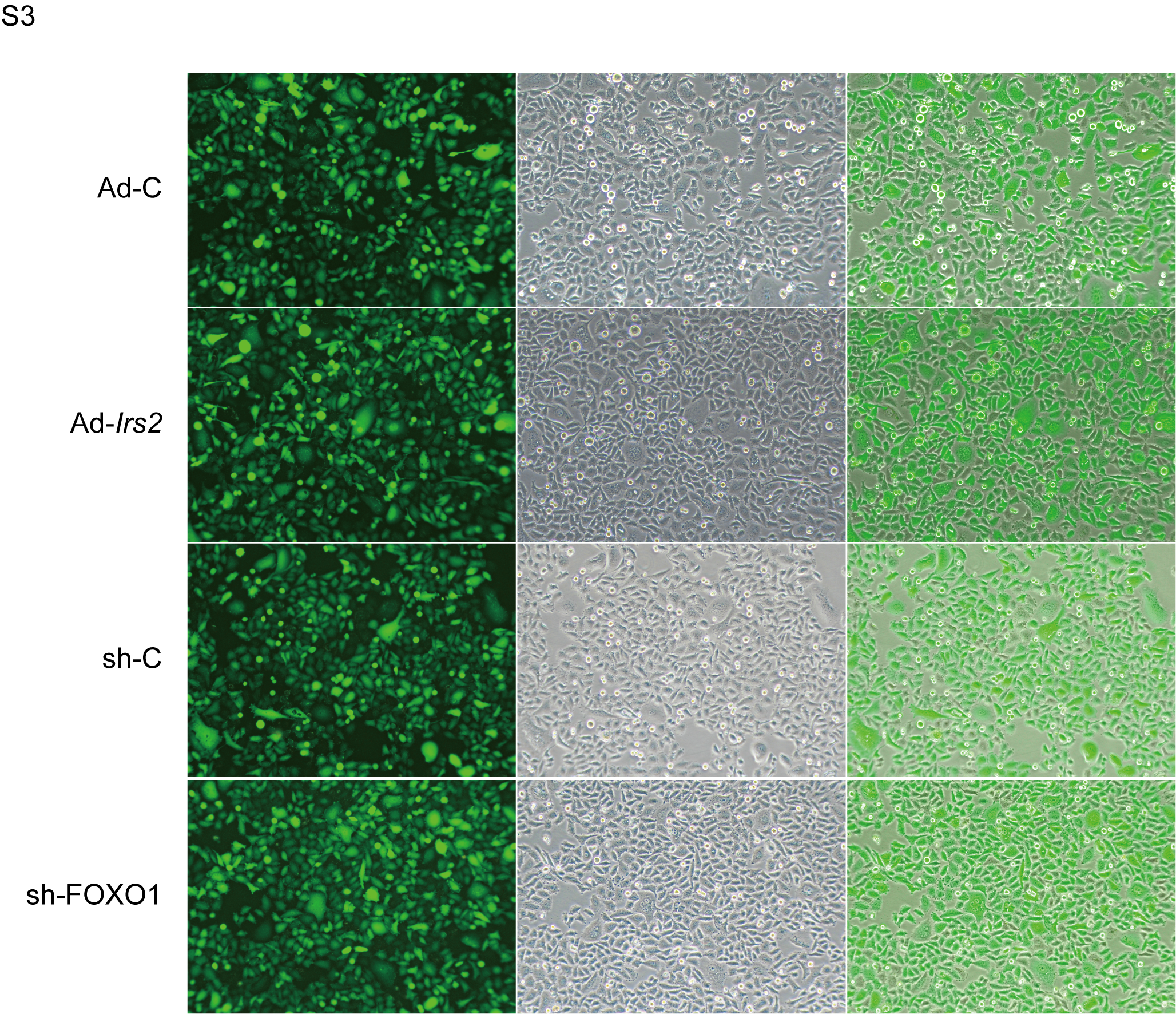

Supplement: Supplementary file 4 — Supplementary Material 4. [file 10020_2025_1346_MOESM4_ESM.png]
